# Paclitaxel-incorporated nanoparticles improve functional recovery after spinal cord injury

**DOI:** 10.3389/fphar.2022.957433

**Published:** 2022-08-05

**Authors:** Xinzhu Zhang, Wu Xiong, Guang Kong, Yushan Zhen, Qiang Zeng, Siming Wang, Sheng Chen, Jun Gu, Cong Li, Kaijin Guo

**Affiliations:** ^1^ Nanjing Medical University, Nanjing, China; ^2^ Department of Orthopedics, The First Affiliated Hospital of Xuzhou Medical University, Xuzhou, China; ^3^ Department of Orthopedics, The First Affiliated Hospital of Nanjing Medical University, Nanjing, China; ^4^ Gusu School, Nanjing Medical University, Suzhou, China; ^5^ Department of Orthopedics, The Affiliated Jiangsu Shengze Hospital of Nanjing Medical University, Suzhou, China; ^6^ Medical College of Jiangsu University, Zhenjiang, China; ^7^ Department of Orthopedics, Xishan People’s Hospital, Wuxi, China

**Keywords:** spinal cord injury, spermine-functionalized acetalated dextran, paclitaxel, chondroitin sulfate proteoglycan inhibition, spinal cord injury

## Abstract

As a worldwide medical problem, spinal cord injury has no clear and effective treatment to improve its prognosis. Hence, new treatment strategies for spinal cord injury with good therapeutic efficacy have been actively pursued. As a new drug loading system, acetal dextran nanoparticles (SAD) have good biocompatibility and biodegradability. Therefore, we designed spermine-functionalized acetal-dextran (SAD) nanoparticles and encapsulated paclitaxel (PCL) into them. This design can ensure the sustained release of paclitaxel in the injured area for 4 days and promote the extension of nerve processes *in vitro*. In our experiment, we found that paclitaxel-loaded SAD nanoparticles (PCL@SAD) decreased the level of chondroitin sulfate proteoglycan in the rat spinal cord injury model, which reduced the scar repair of the injured site and changed the inhibitory environment after spinal cord injury. This reveals that PCL@SAD can effectively protect the injured spinal cord and ultimately improve the functional recovery of the injured spinal cord. One single injection of PCL@SAD shows better therapeutic effect than that of PCL. This study opens an exciting perspective toward the application of neuroprotective PCL@SAD for the treatment of severe neurological diseases.

## 1 Introduction

Spinal cord injury, one of the central nervous system injuries, usually leads to substantial neurological dysfunction and permanent sensory-motor disabilities (French et al., 2007; Jiang et al., 2020; Zhou et al., 2020). Injury to the spinal cord affects approximately 180,000 patients worldwide every year, which has a negative impact on the living quality of patients and increases the financial burden (Fitzhar ris et al., 2014). Spinal cord injury usually leads to the formation of a glial scar and the upregulation of inhibitory factors, such as chondroitin sulfate proteoglycans (CSPGs). These inhibitory factors limit axonal extension and slow down further behavioral recovery (Schwab and Bartholdi, 1996; Burda and Sofronie w, 2014).

The therapy of spinal cord injury is currently focused on rebuilding of functional synapses. To facilitate this rebuilding process, researchers try to improve neurons’ intrinsic growth capacity, for example, decreasing the level of inhibitory factors and removing the extrinsic barriers (Bradbury et al., 2002; Shumsky et al., 2003; Mothe and Tator, 2012). However, only limited and unsatisfactory therapeutic efficacy has been found (Varma et al., 2013). After spinal cord injury, the stabilization of microtubule is indispensable for neuron survival, intracellular signaling, and axonal transport (Hellal et al., 2011). In a spinal cord contusion injury model, Hellal et al. (2011) showed that the continuously intrathecal infusion of paclitaxel (PCL) could improve axonal regeneration and enhance functional recovery. PCL is a clinically approved drug which inhibits mitosis and stabilizes microtubule formation. Because of its poor water solubility, PCL is dissolved in Cremophor EL and ethanol. The used solvents for PCL, especially Cremophor EL, induce peripheral neuropathy (Singla et al., 2002). Therefore, a drug delivery system, which can eliminate Cremophor EL-related side effects and continuously release PCL to the injury site, is expected to improve the clinical outcome of spinal cord injury.

Drug delivery systems are engineering technologies and formulations to control the release of incorporated active ingredients to perform their desired therapeutic effects (Langer, 1998).

SAD has been formulated into nanoparticles using an emulsion approach for biomedical applications, such as gene therapy (Ceaglio et al., 2013) and myocardial infarction treatment (Ferreira et al., 2018). The dual delivery of CHIR99021 and SB431542 by SAD exerted concomitantly anticipated biological effects by stabilizing β-catenin and by preventing translocation of Smad3 to the nucleus of (myo)fibroblasts (Ferreira et al., 2018). SAD nanoparticles co‐loaded with the non‐genotoxic molecule nutlin‐3a and the cytokine granulocyte macrophage colony‐stimulating factor were engineered to induce cancer cell death and create a specific antitumor immune response (Maier and Schwab, 2006; Giger et al., 2010; Bauleth Ramos et al., 2017).

Herein, we are developing a SAD-based drug delivery system to control the release of PCL for the treatment of spinal cord injury. The PCL-loaded SAD (PCL@SAD) is expected to reduce the level of chondroitin sulfate proteoglycans and transform the inhibitory environment after spinal cord injury. Benefiting these effects, PCL@SAD is anticipated to protect the injured spinal cord, and ultimately, to improve functional recovery of the injured spinal cord.

## 2 Results

### 2.1 Optimize the formulation for PCL@SAD

**FIGURE 1:**
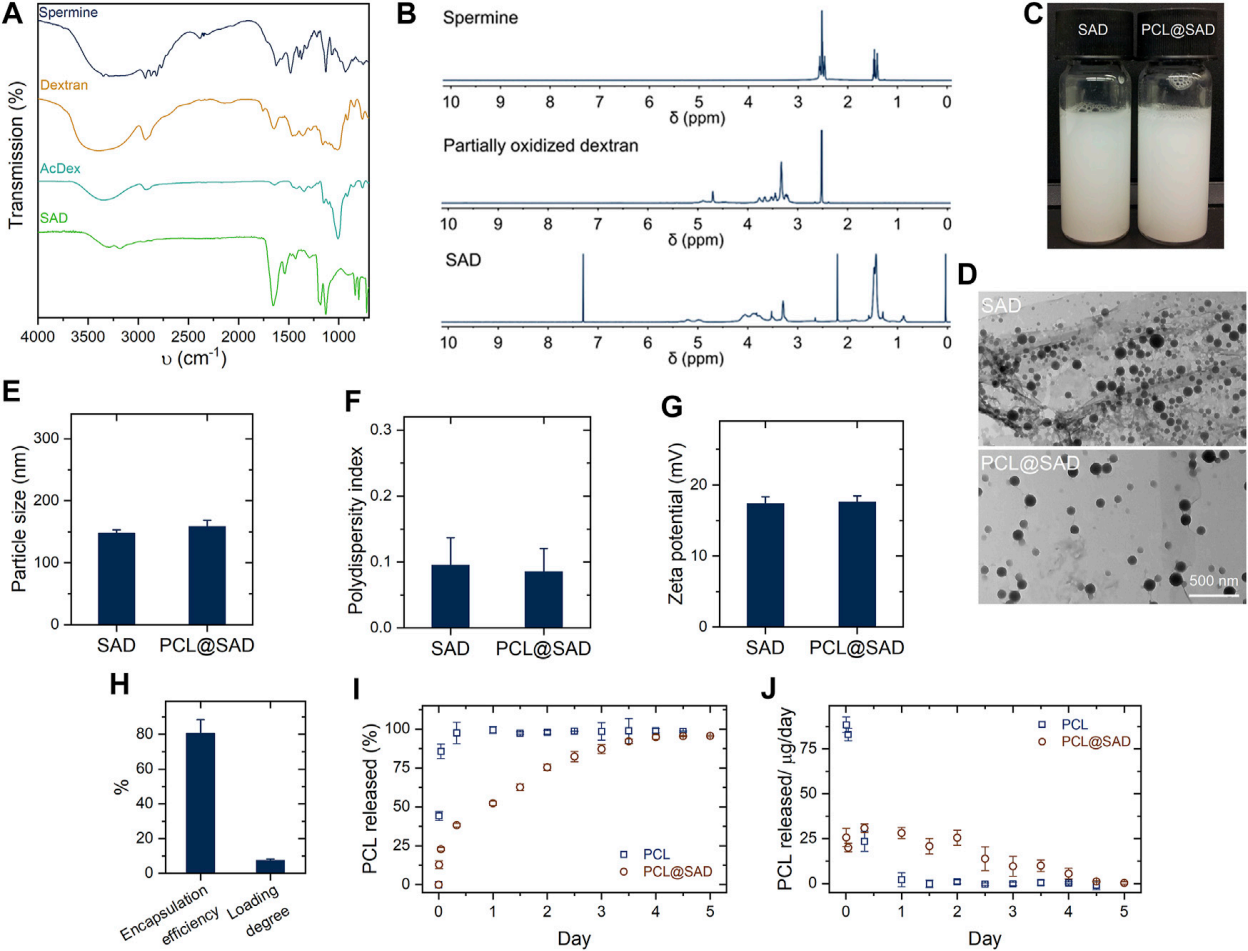
Characterization of SAD nanoparticles. **(A,B)** The Fourier transform infrared **(A)** and nuclear magnetic resonance **(B)** spectra of dextran and SAD. **(C,D)** Representative photos **(C)** and transmission electron microscopy images **(D)** of bare SAD nanoparticles and PCL@SAD nanoparticles. **(E–G)** Average particle size **(E)**, polydispersity index **(F)**, and zeta potential **(G)** of the engineered nanoparticles (*n* = 3). **(H)** Encapsulation efficiency and loading degree of PCL in SAD nanoparticles (*n* = 3). **(I,J)** Cumulative PCL release profiles **(I)** and daily amount of PCL released **(J)** from PCL and PCL@SAD (*n* = 3).

In the presence of an acid catalyst (*p*-toluenesulfonate), the modification in vicinal diols with 2-methoxypropene efficiently transformed the water-soluble dextran into water-insoluble AcDeX (Ceaglio et al., 2013). Fourier transform infrared (Figure 1A) spectra showed a clear intensity decrease in the O-H stretch at 3,325 cm^−1^, which can be ascribed to the reaction of vicinal diols with 2-methoxypropene. In comparison with dextran, the main differences for SAD are the presence of the amide δ(N–H) at 1,537 cm^−1^, suggesting successful functionalization with spermine. Based on the 1H nuclear magnetic resonance (NMR; Figure 1B) spectrum of SAD, the calculated conjugation ratio was approximately 75.1%, corresponding to 45 of 60 glucose units in a dextran (MW 10,000 g/mol) modified by 2-methoxypropene. Figure 1C shows the digital photos of SAD nanoparticles and PCL@SAD nanoparticles, which are both in a milky white color. As illustrated in the representative transmission electron microscopy images (Figure 1D), both bare SAD nanoparticles and PCL@SAD nanoparticles are showing spherical morphology.

PCL@SAD nanoparticles had an average particle size of approximately 158 nm (Figure 1E), which is a little larger than that of bare SAD nanoparticles (147.7 ± 5.5 nm). Both SAD and PCL@SAD showed a narrow size distribution with the polydispersity index close to 0.1 (Figure 1F) and a positively charged surface (approximately 17 mV at pH 7.4; Figure 1G). It seemed that the incorporation of PCL into SAD nanoparticles did not have any impacts on the surface features of ADS nanoparticles. The surface charge values for both SAD and PCL@SAD nanoparticles indicated that PCL was all embedded in the SAD matrix and no free PCL molecules were on the surface of PCL@SAD. The mass fraction of PCL in PCL@SAD nanoparticles was approximately 7.4% with an encapsulation efficiency of around 80.5% (Figure 1H). Moreover, we evaluated the release profile of PCL from PCL@SAD. In the first 8 h, PCL@SAD released approximately 38.2% of PCL; meanwhile, all the PCL released from the bare PCL group (Figure 1I). PCL@SAD achieved complete release of PCL in 3 days (Figure 1J).

### 2.2 *In vitro* cell viability and neuroprotective effect

**FIGURE 2:**
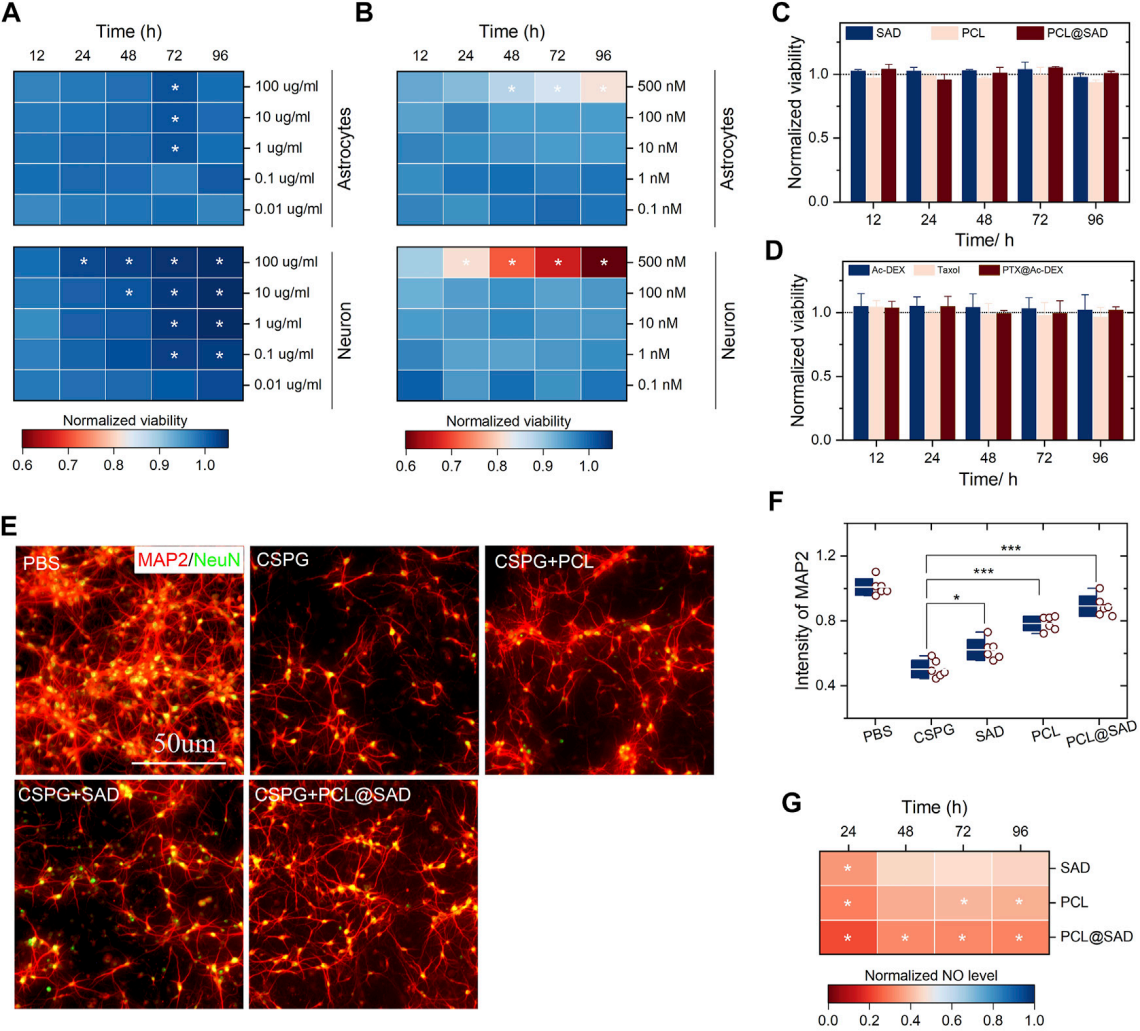
*In vitro* cell viability and neuroprotective effect of PCL@SAD. **(A,B)** The effect of SAD nanoparticles **(A)** and PCL **(B)** on the neuronal and astrocyte viability (*n* = 6). **(C,D)** SAD nanoparticles, PCL, and PCL@SAD did not affect the viability of astrocyte **(C)** and neurons **(D)** (*n* = 6). The concentration of PCL was fixed at 10 nm; the amount of bare SAD nanoparticles was equal to that of PCL@SAD. **(E)** Representative images of axonal regeneration with different formulations after the stimulation with CSPG. The concentration of PCL was fixed at 10 nm; the amount of bare SAD nanoparticles was equal to that of PCL@SAD. **(F)** PCL@SAD, PCL, and SAD all improved the intensity of MAP2, when compared with the PBS group (*n* = 6). **(G)** The effect of PCL@SAD on the release of NO from neurons stimulated by lipopolysaccharide (*n* = 6). The concentration of PCL was fixed at 10 nm; the amount of bare SAD nanoparticles was equal to that of PCL@SAD. ^*^ *p* < 0.05, ^**^ *p* < 0.01, and ^***^ *p* < 0.001.

We evaluated the impact of SAD and PCL (from 0.1 to 500 nm) on the viability of neurons and astrocytes. Regardless of the incubation time (from 12 to 96 h) and nanoparticle concentration (from 0.01 to 100 μg/ml), SAD showed no significant impact on the viability of neurons and astrocytes (Figure 2A). We observed notable neuronal (not astrocyte) viability enhancement in the SAD group with a concentration of 100 μg/ml after 24 h incubation. By extending the incubation time to 96 h, a remarkable neuronal viability increase was observed for SAD nanoparticles with a concentration higher than 0.1 μg/ml. A notable increase in astrocyte viability at SAD concentrations of 1 μg/ml, 10 μg/ml, and 100 μg/ml was observed only at 72 h after incubation. The viability test suggested a neuroprotective effect of SAD nanoparticles, especially at a concentration of 100 μg/ml. At the same time, we found that PCL induced immediate cytotoxicity when the concentration was 500 nm in both neurons and astrocytes (Figure 2B). According to the results of the neuron and astrocyte viability test, we selected the concentration of PCL at 10 nm and evaluated the viability of neurons and astrocytes after incubation with bare SAD nanoparticles, PCL, or PCL@SAD. The results indicated that no obvious cytotoxicity was observed on astrocytes (Figure 2C) and neurons (Figure 2D) in all the groups.

After spinal cord injury, one of the inhibitory factors, chondroitin sulfate proteoglycans (CSPGs), is upregulated, which inhibits axonal extension in the injury site. We verified the effect of PCL on the axonal extension of primary neurons with the presence of CSPG. Neurons were examined by microtubule-associated protein-2 (MAP2; 1:500, mouse IgG1). Figure 2E shows the representative fluorescence images of injured neurons incubated with different formulations. In comparison with the PBS group, CSPG reduced the average intensity of neurite extension by approximately 50% (Figure 2F). In comparison with the CSPG group, bare SAD nanoparticles (*p* < 0.05), PCL (*p* < 0.001), and PCL@SAD (*p* < 0.001) all significantly improved neurite extension. The neurite extension level in the PCL@SAD group was significantly higher than that in the SAD group (*p* < 0.001) and PCL group (*p* < 0.05).

Nitric oxide (NO), an inflammatory mediator, is closely involved in the development of post-traumatic spinal cord cavitation (Sharma et al., 2012). Cytotoxic effects of NO can be ascribed to its interaction with superoxide to form a highly toxic oxidant, peroxynitrite anion (Atwal et al., 2008). Therefore, we quantified the NO level in the primary neurons, which were stimulated by lipopolysaccharide. As shown in Figure 2F, PCL@SAD nanoparticles, regardless of its concentrations, significantly reduced the intensity of NO in neurons. The suppression of NO production by PCL@SAD nanoparticles was maintained over the remainder of the NO assay. Among all three formulations, the highest NO suppression effect was observed for the PCL@SAD group, regardless of the LPS stimulation time (Figure 2G). These results suggested that PCL@SAD can inhibit the release of NO from neurons, which may counteract the relevant secondary inflammatory events in spinal cord injury (Kim et al., 2009; Dickendesher et al., 2012; Popovich et al., 2014; Liddelow et al., 2017).

### 2.3 *In vivo* drug concentration and neuroprotective effect

**FIGURE 3:**
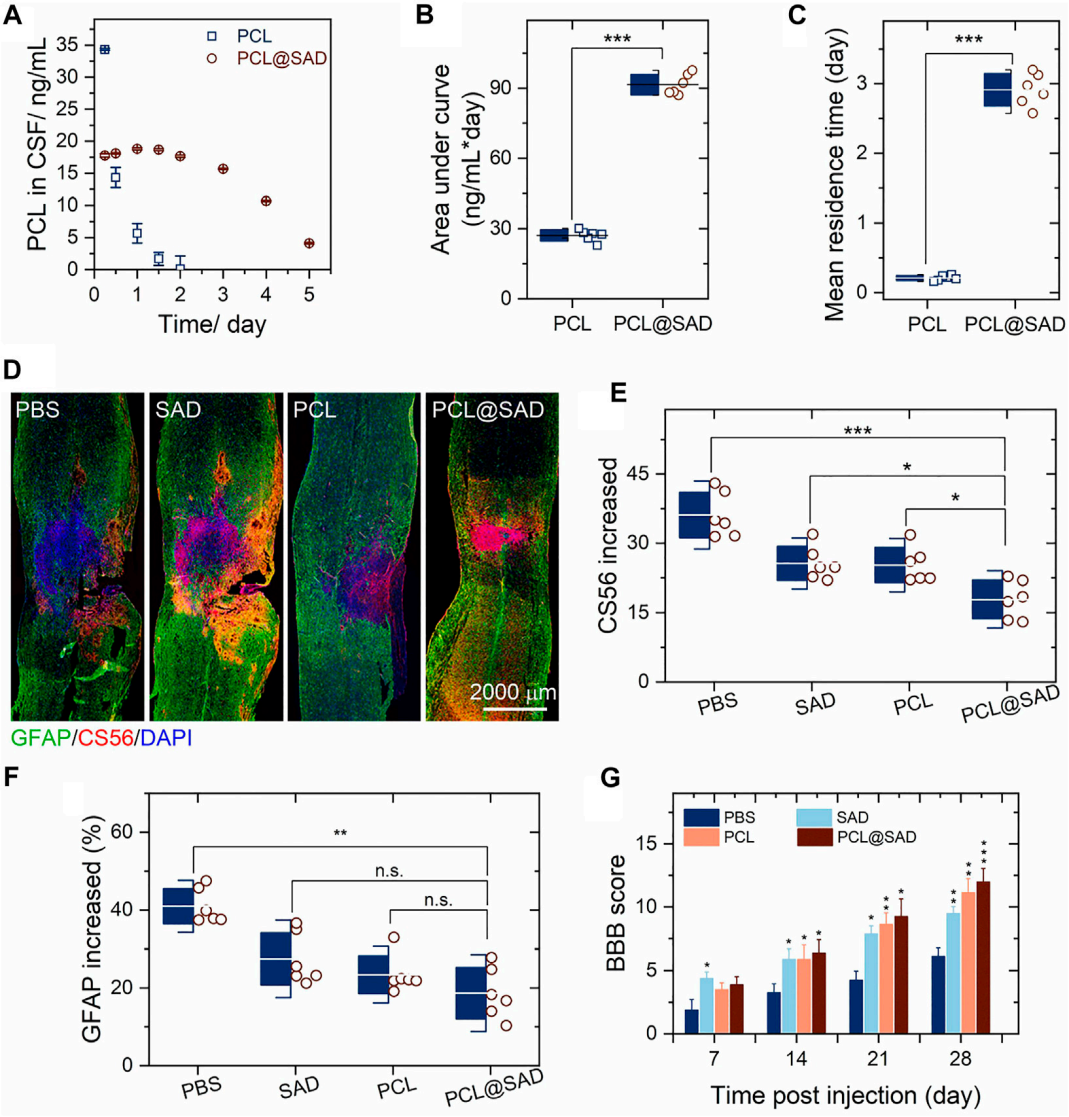
PCL@SAD improves functional recovery after spinal cord injury (*n* = 6). **(A–C)** Concentration of PCL in the cerebrospinal fluid (CSF) as a function of time after intrathecal administration of PCL and PCL@SAD, and the corresponding area under curve (AUC; b) and mean residence time (MRT; c). **(D)** Representative immunohistochemical staining of CS56 (in red) and GFAP (in green) in spinal cord tissues at day 28 post-injury. **(E,F)** Semi-quantification of CS56 **(E)** and GFAP **(F)** intensity and density in the injured spinal cord. **(G)** The effect of PCL@SAD on the BBB grading score of spinal cord injured rats. ^*^ *p* < 0.05, ^**^ *p* < 0.01, and ^***^ *p* < 0.001.

We monitored the concentration of PCL in cerebrospinal fluid (CSF). The PCL content in cerebrospinal fluid was analyzed by liquid chromatography and triple quadrupole mass spectrometry (Li et al., 2016; Liu et al., 2016). On day 1 after the administration of PCL@SAD, the concentration of PCL in CSF was 18.8 ng/ml (Figure 3A), and a slight decrease was noticed every day until reaching a concentration of 10.7 ng/ml on the 4^th^ day after administration (Figure 3A). For the PCL group, drug concentration rapidly declined to about 5.6 ng/ml within 1 day, which may be ascribed to the high permeability of the blood–spinal cord barrier after injury. We also calculated the area under the PCL concentration–time curve (Figure 3B) and the mean residence time of PCL (Figure 3C) in cerebrospinal fluid. The area under the PCL concentration–time curve for PCL@SAD (91.5 ± 4.4 ng/mlday) was significantly (*p* < 0.001) higher than that of PCL (27.1 ± 2.5 ng/mlday). Because of the sustained release of PCL from PCL@SAD nanoparticles, the mean residence time of PCL for PCL@SAD (2.9 ± 0.2 days) was significantly (*p* < 0.001) higher than that of the PCL group (0.2 ± 0.1 day).

To investigate the anatomical basis of the observed locomotor recovery, we examined the density of astrocytes and the level of CSPGs. By staining CS56 to represent CSPGs, we found that the level of CSPGs was significantly reduced in the lesion area in the SAD group. Similar phenomena were also observed in the PCL group and the PCL@SAD group (Figure 3D). When compared with the SAD group and the PCL group, the increase in CSPGs in the lesion area was significantly reduced by PCL@SAD, indicating the promoted extreme axonal regeneration after spinal cord injury (Figure 3E). Simultaneously, we monitored the intensity of glial fibrillary acidic protein (GFAP). Among all the groups, the lowest intensity increase in GFAP was found for PCL@SAD, which indicated the smallest volume of the lesion area (Figure 3F). Therefore, PCL shows a therapeutic effect by promoting axonal extension and reducing the inhibitory molecular deposition in the injured area.

Next, we verified the neuroprotective effect of PCL@SAD in Sprague–Dawley rats undergoing a weight-drop injury toward the thoracic spinal cord. The PBS group served as the control. After traumatic spinal cord injury, motor behavior was assessed by the 21-point Basso, Beattie, and Bresnahan (BBB) locomotor rating scale in an open field. Complete hindlimb paralysis (BBB score = 0) was observed for all four groups on day 1 and day 3 post-injury (data not shown). In comparison to the PBS group, SAD exhibited a faster rising trend in the BBB scores (Figure 3G). This phenomenon is consistent with the study by Jin et al. (Bachelder et al., 2008), in which polymeric microspheres protected neurons from glutamate-induced excitotoxicity. Rats in the SAD group, PCL group, and PCL@SAD group exhibited significant improvement in the BBB score when compared with rats in the PBS group from the second week post-injury (Figure 3G).

After spinal cord injury, upregulated extrinsic factors at the injury site inhibit the functional recovery by limiting axonal outgrowth and preventing reformation of functional synapses (Silver and Miller, 2004; Yiu and He, 2006; Sharma et al., 2012). Therefore, the prevention of actin filament depolymerization is anticipated to enhance axon regrowth (Silver and Miller, 2004; Benson et al., 2005; Yiu and He, 2006; Atwal et al., 2008; Dickendesher et al., 2012). Here, we demonstrated that CSPGs were downregulated using PCL-loaded SAD nanoparticles. As a result, by incorporation of PCL into SAD nanoparticles, low concentration of PCL promoted axonal extension and suppressed CSPGs with a single injection.

## 3 Conclusion

Here, we proposed a promising strategy for spinal cord injury therapy with sustained release of PCL from SAD nanoparticles. After spinal cord injury, the administration of PCL@SAD to the lesion site reduced the inhibitory effect of CSPGs, enhanced neural regeneration, provided neuroprotection to the injured spinal cord, and improved locomotor recovery. After a single injection of PCL@SAD nanoparticles, PCL can be released continuously for 4 days at the injured site without using Cremophor EL and ethanol. Overall, this study opens a new perspective toward the application of dextran-based nanoparticles for the treatment of severe neurological diseases.

## 4 Materials and methods

### 4.1 Synthesis and characterization of spermine-modified acetylated dextran

Spermine-modified acetylated dextran was synthesized as previously described (Ceaglio et al., 2013; Zhang et al., 2021). In brief, dextran was oxidized and then its hydroxyl groups were modified to form the partially oxidized acetalated dextran. After appending spermines, the spermine-modified acetylated dextran (ADS) was obtained.

At first, dextran (Mw 9,000–11,000 g/mol, 5.0 g in 20 ml water) was reacted with sodium periodate (1.1 g) at room temperature for 5 h. The solution was transferred into a cellulose bag (MWCO of 3,500 g/mol) to remove the water-soluble impurities through dialysis against distilled water. After dialysis, the solution was lyophilized to obtain the partially oxidized dextran. Partially oxidized dextran (1.0 g in 10 ml anhydrous DMSO) was reacted with pyridinium *p*-toluenesulfonate (15.6 mg) and 2-methoxypropene (3.4 ml) under a positive pressure of N_2_ for 4 h. After that, triethanolamine (1 ml) was added to quench the reaction and distilled water (pH 8, 100 ml) was added to precipitate the polymer. The partially oxidized acetalated dextran was isolated by centrifuging at 13,000 rpm for 10 min. After vacuum drying oven at 50°C for 12 h, partially oxidized acetalated dextran (1.0 g in 10 ml DMSO) was reacted with spermine (2.0 g) at 50°C for 22 h. Then, NaBH_4_ (2.0 g) was added into the solution and stirred at room temperature for 18 h. After that, ADS was precipitated by adding distilled water (pH 8; 100 ml). The resultant ADS pellet was dried in the vacuum oven at 50°C for 12 h. ^1^H NMR (400 MHz, Bruker, AVANCE NEO) spectra were used for the identification of the chemical structure: spermine: δ 1.46, 2.52, 2.56 (br); partially oxidized dextran: δ 3.22–3.77 (br, dextran) and δ 4.77 (s, −OH); SAD: δ 3.90–4.07, 4.98, 5.20 (br, dextran), δ 3.29 (s, acetal), 3.52 (br, acetal), and δ 1.47, 2.65 (s, spermine).

In the FTIR spectrum of spermine, characteristic absorption band peaks at 3,348, 3,288 cm-1 (-NH_2_), 2,928, 2,873, 2,818 cm^−1^ (-CH_2_-, stretching), and 1,570 cm^−1^ (-NH-, bending). FTIR spectra showed a clear intensity decrease in the O-H stretch at 3,390 cm^−1^ when comparing AcDeX with dextran, which can be ascribed to the modifying hydroxyl groups of dextran with 2-methoxypropene. The spermine-functionalized acetylated dextran was confirmed by the presence of a broad stretching peak at 3,298 cm^−1^ and 3,180 cm^−1^ (-NH_2_, spermine).

### 4.2 Preparation of SAD and PCL@SAD nanoparticles

SAD nanoparticles were prepared using the micro-precipitation method. In brief, SAD ethanol solution (20 mg/ml) was dropwise added into the Poloxamer-407 solution (1%, w/v) under stirring. The nanosuspension was collected by centrifugation (20,000 g × 3 min). The obtained nanoparticles were washed twice with 1 × PBS. To prepare PCL@SAD nanoparticles, PCL (2 mg/ml) was added to the SAD solution. After that, the ethanol solution containing SAD and PCL was dropwise dripped into the poloxamer-407 solution (5 ml; 1%, w/v) under stirring to form the PCL@SAD nanoparticles.

### 4.3 Characterization of SAD and PCL@SAD nanoparticles

The morphology of nanoparticles was evaluated using a transmission electron microscope (TEM, Jeol 1,400). The TEM samples were prepared by depositing 2 µl of the nanoparticle suspensions (1.0 mg/ml) onto carbon-coated copper grids (300 mesh; Electron Microscopy Sciences, United States). Samples were blotted away after 5 min incubation, and the grids were then washed twice with distilled water and air-dried prior to imaging.

The particle size and surface zeta (ζ) potential of the nanoparticles were analyzed using dynamic light scattering with a ZetaSizer Ultra (Malvern Instruments Ltd., United Kingdom). For measuring the particle size, 1 ml of the sample was put in a disposable polystyrene cuvette (Sarstedt AG and Co., Germany), and the determination was recorded as the average of three measurements. For measuring the ζ-potential, the samples were measured using disposable folded capillary cells (DTS1070, Malvern, United Kingdom) at pH 7.4 after proper dilution.

PCL concentration was determined using Agilent 1,260 (Agilent Technologies, Santa Clara, CA, United States). A C_18_ column (Agilent Technologies) was used as the stationary phase and set at 30°C. The mobile phase was composed of water and acetonitrile (15:85, v/v), and the flow rate was 1.0 ml/min. The injection volume was 50 μl, and the detection wavelength was 227 nm.

The encapsulation efficiency was calculated by measuring the added amount of PCL and the amount in the nanoparticles. The loading degree of PCL was calculated by measuring the amount of PCL in the nanoparticles and the total weight of the nanoparticles. The release of PCL from PCL@SAD was determined by placing PCL@SAD into the CSF (10 ml) to simulate the central nervous system environment at 37°C.

The drug and entrapment efficiency were calculated from the following equations:

$$Drug\;loading\;degree=\frac{mass\;of\;loaded\;cargoes}{mass\;of\;obtained\;composites}\times 100\%.$$

$$Encapsulation\;efficiency=\frac{actual\;mass\;of\;encapsulated\;cargoes}{initial\;mass\;of\;cargoes\;added}\times 100\%.$$

### 4.4 *In vitro* study

#### 4.4.1 Culture of neurons and astrocytes

According to an established protocol (Takei et al., 1998), primary neurons were collected from embryonic (E16–18) Sprague–Dawley (SD) rats. In brief, cerebral cortices were isolated and dissociated with trypsin (0.25%, w/v; Thermo Fisher Scientific, United States) for 20 min. Neurons were seeded at a density of 1 × 10^5^/ml for immunofluorescent staining in 24-well culture plates and 5 × 10^4^/ml for viability assays on 96-well culture plates. Neurons were maintained in a fresh neurobasal medium (Thermo Fisher Scientific) containing 2% B27 (2%, w/v; Thermo Fisher Scientific), 1% glutamine (Thermo Fisher Scientific), 100 IU/ml penicillin, and 100 mg/ml streptomycin. Half of the medium was changed every 3 days. After 5 days of cell culture, the obtained neurons were examined by microtubule-associated protein-2 (MAP2; 1:500, mouse IgG1; Abcam, United States) under a fluorescence microscope.

Primary astrocytes were obtained from ScienCell (ScienCell Research Laboratories). They were harvested into poly-d-lysine-coated culture plates and maintained in an astrocyte medium supplemented with 2% fetal bovine serum, 1% astrocyte growth supplement (ScienCell Research Laboratories), and 1% penicillin/streptomycin solution (ScienCell Research Laboratories). Half of the medium was changed every other day. Both neurons and astrocytes were maintained in a standard incubator at 37°C with an atmosphere of 5% (v/v) CO_2_ and 95% relative humidity.

#### 4.4.2 Cell viability assay

The viability of neurons and astrocytes was evaluated with a CCK-8 assay (Dojindo, Kumamoto, Japan). After incubation, the wells were rinsed three times with 1 × PBS. Then, CCK-8 solution (10 μl; 1:10 diluted) in the neurobasal medium was added and incubated for 2 h at 37°C. The optical absorbance was measured at 450 nm using an absorbance microplate reader (ELx800, Bio-Tek, United States).

### 4.5 *In vivo* study

#### 4.5.1 Construction of spinal cord injury model

All procedures were conducted according to the Guidelines for the Care and Use of Laboratory Animals and were approved by the Animal Care and Use Committee of Nanjing Medical University. Female rats (170–220 g) were anesthetized with chloral hydrate (350 mg/kg of body weight). After animals became unresponsive, skin and muscle were opened on the back to expose the vertebral column. A T10 laminectomy was performed, and the exposed dorsal surface of the cord was subjected to weight-drop impact using a 10 g rod (2.5 mm in diameter; RWD Life Science Corp, C4p01-001, China) dropped from a height of 12.5 mm. The muscles were sutured immediately after formulation administration and then the skin was closed. The bladders of animals were manually voided three times per day until the reflexive control of bladder function was restored.

#### 4.5.2 Administration of bare SAD nanoparticles, PCL, and PCL@SAD

Rats were randomly assigned to the following four groups: PBS, SAD, PCL, and PCL@SAD. After loading into a sterilized 26G Hamilton syringe, PBS, SAD, or PCL@SAD was injected approximately 1 mm rostral and caudal to the lesion epicenter. After each injection, the needle was maintained for an additional 2 min to minimize the leakage of injected formulations.

#### 4.5.3 Assessment of locomotor capacity

Locomotion recovery after SCI was scored according to the BBB open-field 21-point locomotion rating scale (Cohen et al., 2011; Zeng et al., 20192019). The movements of hindlimbs were assessed weekly by two independent examiners blinded to the treatment regimen.

#### 4.5.4 Spinal cord tissue immunofluorescence

Primary antibodies used in this study included GFAP (1:1,000, rabbit IgG1; Abcam) and CS56 (1:350, mouse IgG1; Abcam). The secondary antibody used was Cy3-or FITC-conjugated secondary antibody (1:200, Jackson ImmunoResearch, United States). First, spinal cord sections on day 28 post-injury were permeabilized for 30 min in Triton X-100 PBS solution (0.3%, w/v) and then blocked with natural goat serum PBS solution (10%, v/v). The specimens were incubated with primary antibodies overnight at 4°C, triple-washed with PBS, and then incubated with a secondary antibody for 2 h at room temperature. After triple washing with PBS, nuclei were stained with DAPI, and fluorescent images were taken. For each side, the lesion was identified as the area lacking staining. We selected six different areas near the traumatic lesion as the near-injury area. Six different areas at least 10 mm distance from the traumatic lesion were chosen as far-injury areas. The average intensity of NF200, GFAP, and CS56 was measured with ZEN lite software. Data are expressed as the percentage of intensity increase or decrease in the near-injury area compared to the far-injury area. All images were taken at the same exposure time and conditions.

#### 4.5.5 Cerebrospinal fluid sampling

We collected cerebrospinal fluid with a syringe equipped with a 25G disposable needle (0.5 mm × 20 mm) as described previously (Ceaglio et al., 2013). At first, we washed the rat’s head with soap and water, removed the hair with a shaving blade, and fixed the head at an angle of about 135°. Then, the occipital crest was located, and the needle was carefully inserted from the caudal end at 30° to the body in the muscle gap at 3 mm below the occipital crest. The tip of the needle was slowly moved to the cerebellum until a depth of about 0.5 cm. When the needle touched the yellow ligament, a sense of resistance was felt followed by a hollow feeling as the needle pierced the yellow ligament. At this point, the tip of the needle reached the cerebellum into the pool. The needle was immediately retracted and 80–100 µl cerebrospinal fluid was slowly extracted in less than 1 min. The obtained samples were immediately centrifuged to remove any blood and PCL@SAD nanoparticles that might be present, and then stored at −80°C before analysis.

#### 4.5.6 Quantification of PCL in CSF

The PCL concentration in cerebrospinal fluid was analyzed by a liquid chromatography–tandem mass spectrometry. The liquid chromatography system composed of a binary pump (LC-30AD), an autosampler (SIL-30AC), a column oven (CTO-20A), a system controller (CBM-20A), and a degasser (DGU-20A). Mass spectrometric analysis was performed using an AB SCIEX API6500^+^ triple-quadrupole instrument (Ontario, Canada) with an electrospray ionization interface. Data acquisition and the control system were created by using Analyst 1.6.2 software from AB SCIEX. Chromatographic separation was performed on a C18 column (XBridge UPLC BEH; 2.1 mm × 50 mm, 2.5 µm), with water as mobile phase A (0.025% formic acid and 1 mm ammonium acetate) and acetonitrile as mobile phase B (0.025% formic acid and 1 mm ammonium acetate). The column was eluted at a flow rate of 0.8 ml/min in a gradient program consisting of 10% phase B (0–0.2 min), 10–95% phase B (0.2–0.6 min), 95% phase B (0.6–1.20 min), 95–10% phase B (1.20–1.21 min), and 10% phase B (1.21–1.80 min). The retention time for PCL and internal standard (Glipizide) was 0.85 and 0.80 min, respectively. The precursor and product–ion pairs were m/z 876.4→591.1 for PCL and m/z 446.2→321.1 for glipizide. An aliquot of 6 µl sample was diluted with methanol solution (24 μl; 80%, v/v). For protein precipitation, glipizide internal standard (200 μl; 50 ng/ml in acetonitrile) was added. The mixture was vortexed for 10 min at 750 rpm and centrifuged at 6,000 rpm for 10 min. An aliquot of 2 µl supernatant was injected for analysis.

#### 4.5.7 Statistical analysis

Data were expressed as mean ± standard deviation for at least six independent experiments. Multiple-group comparisons were made using a one-way analysis of variance. Two-group comparisons were tested with Student’s t-test; * represented a *p* value <0.05, ** represented a *p* value <0.01, and *** represented a *p* value <0.001.

## Data Availability

The original contributions presented in the study are included in the article/Supplementary Material; further inquiries can be directed to the corresponding authors.
