# Corrigendum: Paclitaxel-incorporated nanoparticles improve functional recovery after spinal cord injury

**DOI:** 10.3389/fphar.2024.1451951

**Published:** 2024-08-01

**Authors:** Xinzhu Zhang, Wu Xiong, Guang Kong, Yushan Zhen, Qiang Zeng, Siming Wang, Sheng Chen, Jun Gu, Cong Li, Kaijin Guo

**Affiliations:** ^1^ Nanjing Medical University, Nanjing, China; ^2^ Department of Orthopedics, The First Affiliated Hospital of Xuzhou Medical University, Xuzhou, China; ^3^ Department of Orthopedics, The First Affiliated Hospital of Nanjing Medical University, Nanjing, China; ^4^ Gusu School, Nanjing Medical University, Suzhou, China; ^5^ Department of Orthopedics, The Affiliated Jiangsu Shengze Hospital of Nanjing Medical University, Suzhou, China; ^6^ Medical College of Jiangsu University, Zhenjiang, China; ^7^ Department of Orthopedics, Xishan People’s Hospital, Wuxi, China

**Keywords:** spinal cord injury, spermine-functionalized acetalated dextran, paclitaxel, chondroitin sulfate proteoglycan inhibition, spinal cord injury

In the published article, there was an error regarding the **Affiliations** for Kaijin Guo. As well as having affiliation 2, they should also have 1 Nanjing Medical University, Nanjing, China.

The authors apologize for this error and state that this does not change the scientific conclusions of the article in any way. The original article has been updated.

